# Dopamine receptor D1- and D2-agonists do not spark brown adipose tissue thermogenesis in mice

**DOI:** 10.1038/s41598-020-77143-6

**Published:** 2020-11-19

**Authors:** Francesca-Maria Raffaelli, Julia Resch, Rebecca Oelkrug, K. Alexander Iwen, Jens Mittag

**Affiliations:** 1 Department of Molecular Endocrinology, Institute for Endocrinology and Diabetes, University of Lübeck, Ratzeburger Allee 160, 23562 Lübeck, Germany; 2 Department of Internal Medicine I, University Medical Center Schleswig-Holstein, University of Lübeck, Ratzeburger Allee 160, 23562 Lübeck, Germany

**Keywords:** Fat metabolism, Homeostasis, Obesity

## Abstract

Brown adipose tissue (BAT) thermogenesis is considered a potential target for treatment of obesity and diabetes. In vitro data suggest dopamine receptor signaling as a promising approach; however, the biological relevance of dopamine receptors in the direct activation of BAT thermogenesis in vivo remains unclear. We investigated BAT thermogenesis in vivo in mice using peripheral administration of D1-agonist SKF38393 or D2-agonist Sumanirole, infrared thermography, and in-depth molecular analyses of potential target tissues; and ex vivo in BAT explants to identify direct effects on key thermogenic markers. Acute in vivo treatment with the D1- or D2-agonist caused a short spike or brief decrease in BAT temperature, respectively. However, repeated daily administration did not induce lasting effects on BAT thermogenesis. Likewise, neither agonist directly affected *Ucp1* or *Dio2* mRNA expression in BAT explants. Taken together, the investigated agonists do not seem to exert lasting and physiologically relevant effects on BAT thermogenesis after peripheral administration, demonstrating that D1- and D2-receptors in iBAT are unlikely to constitute targets for obesity treatment via BAT activation.

## Introduction

Obesity and its metabolic consequences like type 2 diabetes have spread in recent years to become a worldwide pandemic^1,2^. It is therefore highly important to elucidate the complex pathways of energy homeostasis in order to develop approaches to counteract these dysregulations of metabolism. In addition to advocating individual responsibility regarding energy intake and expenditure, which may present particularly difficult in highly obese individuals^3,4^, an endogenous increase of energy expenditure proposes a potential strategy. Regarding this, activation and recruitment of brown adipose tissue (BAT) thermogenesis have been discussed recently^5–8^.

Brown adipocytes have the unique ability to combust energy from metabolic substrates directly into heat^9,10^, making this tissue a promising strategic target to treat metabolic disorders. Therefore, a safe and long-lasting way of BAT thermogenesis activation would be required, and consequently, knowledge of the underlying modes of activation is crucial to assess its true potential for safe exploitation.

The catecholamine neurotransmitter norepinephrine (NE) is a key player in BAT thermogenesis activation^11^. NE binds to the adrenergic receptors α_1_, α_2_, and β, which all exist in BAT^12^. Nevertheless, the interaction between NE and β3-adrenoceptors (ADRB3) is the most relevant to induce thermogenesis in mature brown adipocytes^6,13,14^. Binding of NE to the G_s_-coupled ADRB3 induces an adenosine cyclase (AC)- and cyclic adenosine monophosphate (cAMP)-mediated cascade that increases expression of uncoupling protein 1 (UCP1), which in turn bypasses oxidative phosphorylation in the respiratory chain and causes heat dissipation^15,16^. Interestingly, NE simultaneously binds to G_i_-coupled α_2_-adrenergic receptors and inhibits AC activity, counteracting ADRB3-mediated activation of BAT thermogenesis^17^. The purpose of this counter regulation is not understood to date, but underlines that endogenous neurotransmitter-receptor relationships are complex and not always specific^18–20^. To dissect the significance of distinct receptor-mediated pathways on BAT thermogenesis, they need to be studied separately, e.g., by using specific receptor agonists instead of the endogenous ligand.

The catecholamine neurotransmitter dopamine is the direct metabolic precursor of NE, also binding to G-protein-coupled receptors^21,22^. Dopamine receptors are categorized into D1-like and D2-like dopamine receptors^23^. D1-like dopamine receptors (D1 and D5) are G_s_-coupled receptors and stimulate cAMP synthesis, whereas D2-like dopamine receptors (D2, D3, and D4) are G_i_-coupled and inhibit cAMP synthesis upon ligand-binding^24,25^. Interestingly, it was suggested that dopamine—structurally highly similar to NE—also modulates BAT thermogenesis in rodents, due to comparable mode of action^26,27^. Additional studies have shown that dopamine and the D1-agonist SKF38393 increase thermogenesis and mitochondrial mass of brown adipocytes in vitro^28^. However, whether dopamine receptor signaling directly affects BAT thermogenesis in vivo has not been studied. Here, using primary mouse BAT explants and mouse models, we characterize the direct effects of the D1-agonist SKF38393^29^ and D2-agonist Sumanirole^30,31^ on BAT thermogenesis in detail. Our results show, that neither the D1-agonist, nor the D2-agonist have a sustained BAT-specific effect on thermogenesis upon direct application ex vivo or peripheral administration in vivo.

## Results

### Ex vivo effects of dopamine receptor agonists on mRNA expression of key thermogenic markers in iBAT explants

**Figure 1:**
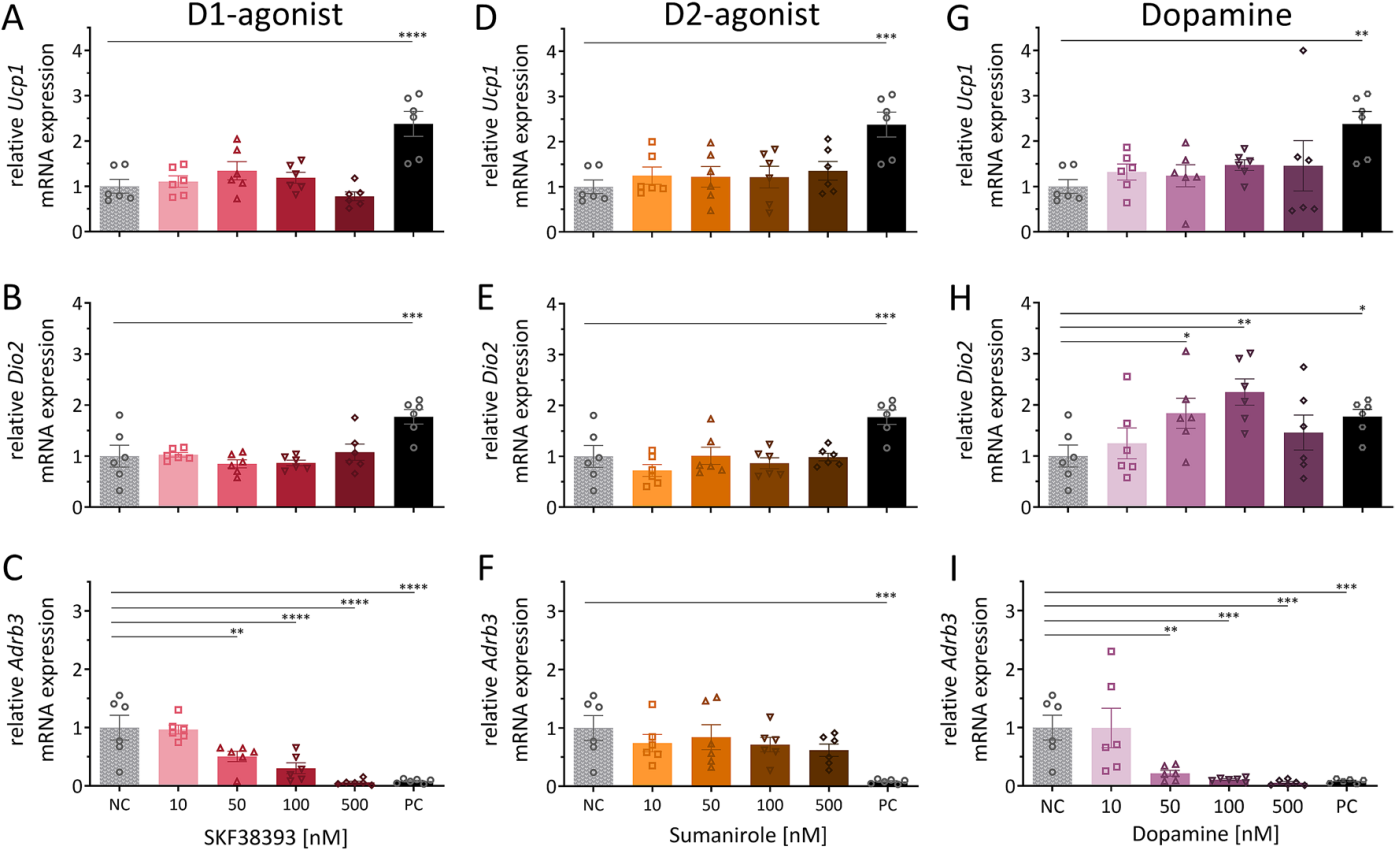
Ex vivo effects of dopamine receptor agonists on mRNA expression of key thermogenic markers in iBAT explants. mRNA expression of key thermogenic markers *Ucp1* (**A**,**D**,**G**), Dio2 (**B**,**E**,**H**), and *Adrb3* (**C**,**F**,**I**) after 24 h treatment of iBAT explants from wild type mice with D1-agonist SKF38393 (left), D2-agonist Sumanirole (middle) or dopamine (right). *NC* negative control (vehicle), *PC* positive control (ADRB3-agonist CL316,243; 1 µM). Data are expressed as mean ± SEM. Conditions were compared to the NC using 1 W ANOVA (uncorrected Fisher’s LSD). **P < 0.01; ***P < 0.001; ****P < 0.0001; n = 6.

To identify possible direct effects of dopamine receptor agonists on thermogenesis, we investigated mRNA expression of key thermogenic markers in interscapular BAT (iBAT) tissue explants. We validated this ex vivo experimental model using the ADRB3-agonist CL316,243 as a positive control (PC). As expected, the PC increased *Ucp1* and *Dio2* mRNA expression and decreased mRNA expression of *Adrb3* itself (Fig. 1A–I). However, treatment with D1-agonist SKF38393 (Fig. 1A,B) or D2-agonist Sumanirole (Fig. 1D,E) did not change mRNA expression of thermogenesis markers *Ucp1* or *Dio2* at any of the tested concentrations. Interestingly, the D1-agonist at 50–500 nM significantly decreased *Adrb3* mRNA expression similarly to the PC (Fig. 1C). The D2-agonist had no effect on *Adrb3* mRNA expression (Fig. 1F). In addition to the D1- and D2-agonist, we investigated dopamine itself to gather insight on the physiological effect of dopamine on iBAT thermogenesis markers ex vivo. As seen with the D1-agonist, dopamine did not change *Ucp1* and significantly reduced *Adrb3* (50–500 nM) mRNA expression (Fig. 1G,I). Interestingly, dopamine also increased *Dio2* mRNA expression at 50 and 100 nM but not 500 nM (Fig. 1H).

### Acute in vivo changes of iBAT temperature after a single i.p. injection of dopamine receptor agonists in wild type mice

**Figure 2:**
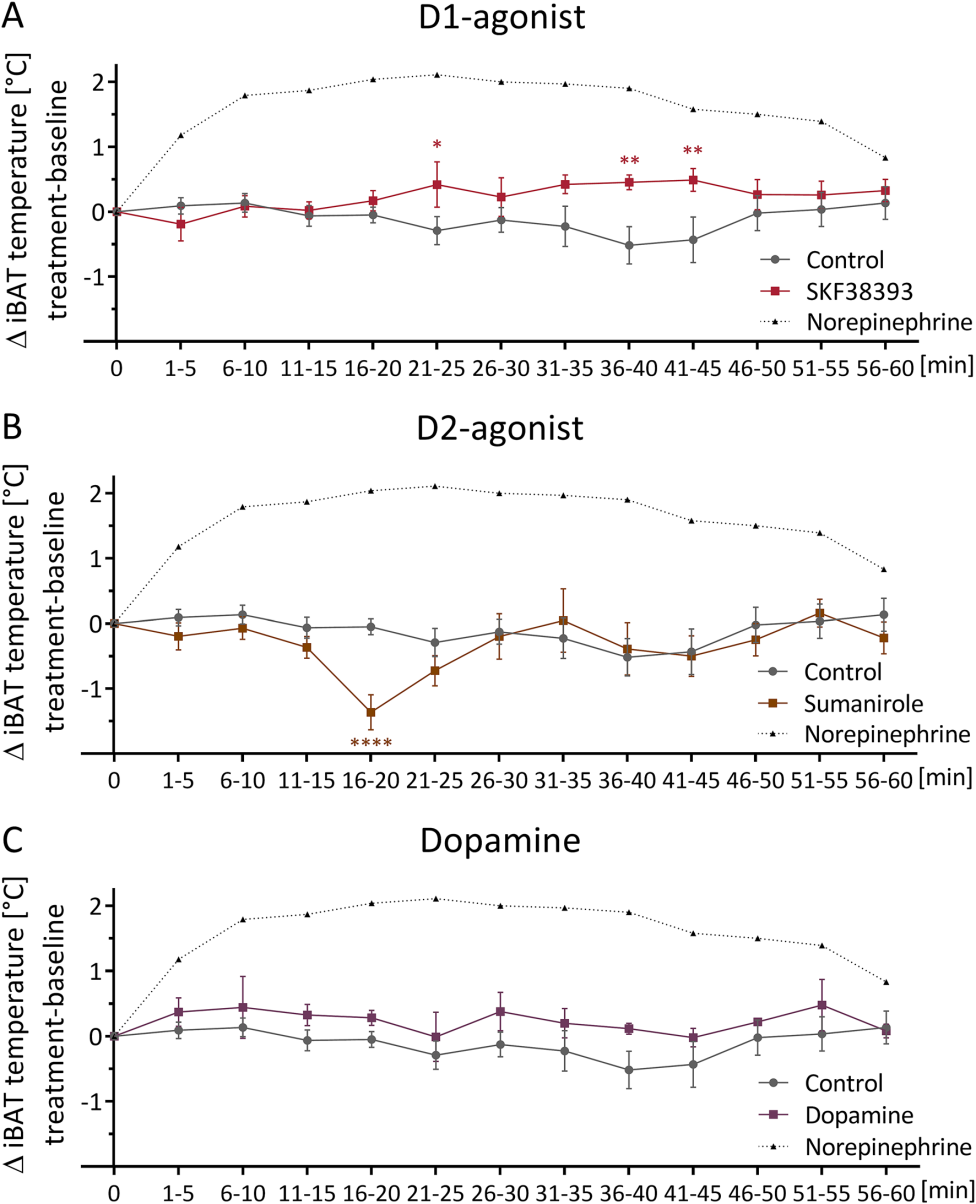
Acute in vivo changes of iBAT temperature after a single i.p. injection of dopamine receptor agonists in wild type mice. ΔiBAT temperature after a single i.p. injection of D1-agonist SKF38393 (10 mg/kg) (**A**), D2-agonist Sumanirole (3.2 mg/kg) (**B**), or dopamine (100 µg/kg) (**C**), compared to injection of NaCl (Control), as determined by infrared video thermography. iBAT temperature following treatment was normalized to average baseline iBAT temperature of minutes − 10 to − 1 (not depicted). As a positive control example, the iBAT temperature change response of a representative animal injected with Norepinephrine is plotted as a black dotted line. All other data points are expressed as mean ± SEM. Groups were compared using 2 W RM ANOVA with Holm-Sidak’s multiple comparison test. *P < 0.05; **P < 0.01; ****P < 0.0001; n = 6.

To capture rapid effects of the D1- and D2-agonist on iBAT thermogenesis, we continuously and non-invasively measured iBAT temperature in wild type mice up until 1 h after i.p. injection using infrared video thermography. While the D1-agonist significantly increased iBAT temperature for a total of 15 min between minutes 20 and 45 compared to baseline (Fig. 2A), the D2-agonist significantly decreased iBAT temperature from minute 15 to 20 compared to baseline (Fig. 2B). Notably, both treatments only caused very brief and transient effects that did not result in long-lasting changes in iBAT temperature. Additionally, we investigated dopamine itself on iBAT temperature, which had no acute effect (Fig. 2C). Our infrared setup was validated using NE as a positive control reference, which is depicted for comparison (Fig. 2A–C).

### In vivo effects of dopamine receptor agonists on wild type mice during daily i.p. injections for 7 days

**Figure 3:**
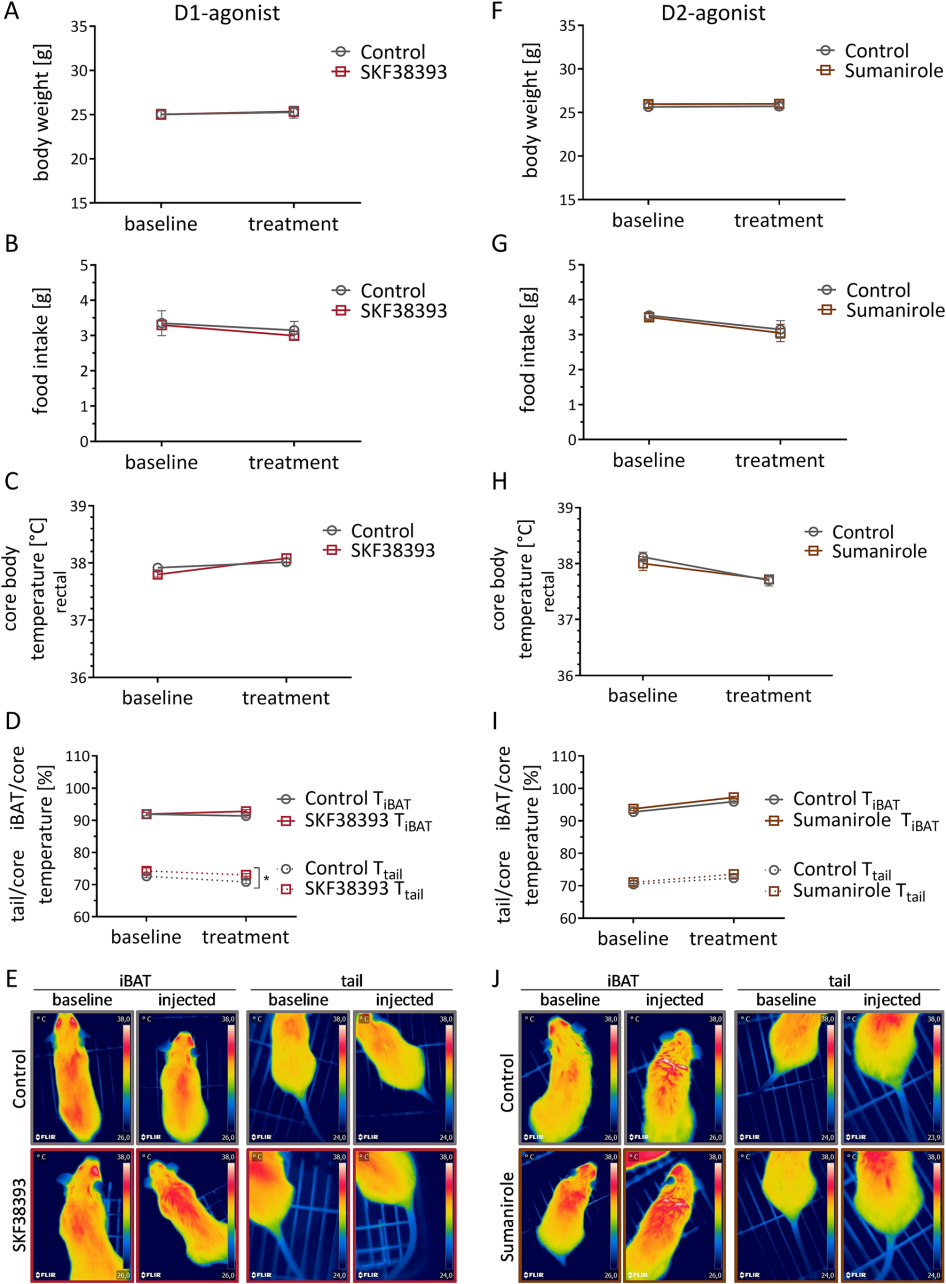
In vivo effects of dopamine receptor agonists on wild type mice during daily i.p. injections for 7 days. Animals treated with D1-agonist SKF38393 (10 mg/kg) (**A**–**E**) or D2-agonist Sumanirole (3.2 mg/kg) (**F**–**J**) compared to respective control groups which received NaCl only. Monitored mean body weight (**A**,**F**), food intake (**B**,**G**), and rectally measured core body temperature (**C**,**H**) before and after beginning of treatment, respectively. iBAT and tail base temperature normalized over rectally measured core body temperature (**D**,**I**), each measured before and after treatment. Representative infrared thermography images of animals treated with D1-agonist SKF38393 (10 mg/kg) (**E**) or D2-agonist Sumanirole (3.2 mg/kg) (**J**) compared to their respective control groups. Data are expressed as mean ± SEM. Groups were compared using 2WA with Bonferroni’s multiple comparison test. *P < 0.05; n = 6.

By administering daily i.p. injections of the D1- or D2-agonist, we tested whether repetitive treatment would lead to more pronounced and lasting effects on iBAT thermogenesis. However, neither repeated D1- nor D2-agonist injections had a significant effect on body weight (Fig. 3A,F), food intake (Fig. 3B,G), core body temperature (Fig. 3C,H), or normalized iBAT temperature (Fig. 3D,E,I,J). Interestingly, the normalized tail base temperature increased mildly during treatment with the D1-agonist compared to its respective control (Fig. 3D,E), while no effect was seen with the D2-agonist (Fig. 3I,J). Moreover, pulse and blood pressure were not affected by either treatment (Supplementary Fig. S1A,D).

### In vivo effects of dopamine receptor agonists on fat depots of wild type mice after daily i.p. injections for 7 days

**Figure 4:**
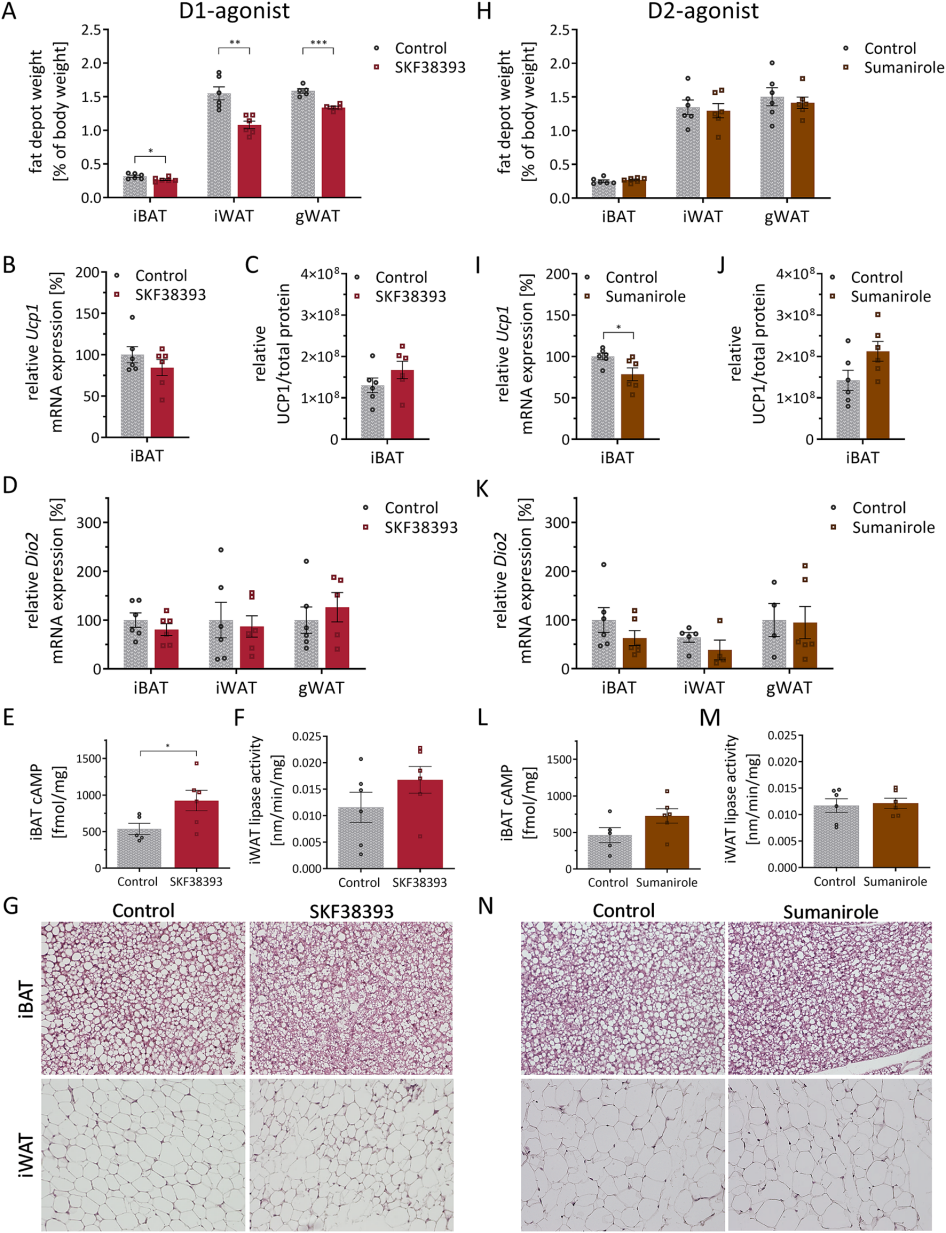
In vivo effects of dopamine receptor agonists on fat depots of wild type mice after daily i.p. injections for 7 days. Fat depot weights (**A**,**H**), relative expression of *Ucp1* mRNA (**B**,**I**)and UCP1 protein (**C**,**J**) in iBAT, *Dio2* mRNA expression in iBAT, iWAT, and gWAT (**D**,**K**), cAMP in iBAT (**E**,**L**), lipase activity in iWAT (**F**,**M**), and representative images of histological H&E staining of iBAT and iWAT (20 × magnification) (**G**,**N**); all determined or obtained after organ collection on day 7 of treatment with either D1-agonist SKF38393 (10 mg/kg) (left) or D2-agonist Sumanirole (3.2 mg/kg) (right). Data are expressed as mean ± SEM. Groups were compared using two-tailed t-tests. *P < 0,05; **P < 0,01; n = 6.

In addition to the physiological parameters, we investigated adipose tissue homeostasis. Repetitive treatment with the D1-agonist resulted in significantly decreased weight of the collected fat depots, namely iBAT, inguinal and gonadal white adipose tissue (iWAT and gWAT) (Fig. 4A); while the weight of other organs or muscles was not affected (Supplementary Fig. S1B,C). Expression of the key thermogenic marker Ucp1 in iBAT did not change on mRNA (Fig. 4B) or protein level (Fig. 4C, Supplementary Fig. S2A). *Ucp1* mRNA expression in iWAT was close to background in all conditions (data not shown) indicative of no WAT browning, while *Dio2* mRNA expression in iBAT, iWAT and gWAT was also not altered by the D1-agonist (Fig. 4D). However, we found cAMP in iBAT of these mice markedly increased (Fig. 4E), indicating that the D1-agonist had some molecular effect on the tissue—presumably by stimulatory G_s_ protein coupled receptors. Lipase activity in iWAT did not increase significantly compared to the control group (Fig. 4F), but histological H&E staining displayed more multilocular fat droplets in brown adipocytes of iBAT and reduced size of white adipocytes in iWAT of D1-agonist-treated mice (Fig. 4G). Treatment with the D2-agonist resulted in no significant effects on organ or muscle weights (Fig. 4H, Supplementary Fig. S1E,F), *Dio2* mRNA expression (Fig. 4K), cAMP protein (Fig. 4L), or lipase activity (Fig. 4M). Further, there were no visible changes in the histological composition of iBAT or iWAT (Fig. 4N). Interestingly, we did see a significant reduction of *Ucp1* mRNA expression in iBAT of D2-agonist-treated mice (Fig. 4I). However, this effect did not translate to altered protein level (Fig. 4J, Supplementary Fig. S2B). We also analyzed protein expression of the five complexes of oxidative phosphorylation (OXPHOS) in iBAT, which were all together not changed by D1- or D2-agonist-treatment (Supplementary Fig. S2A–D), indicating no major metabolic alterations in BAT oxidative capacity. In addition, repeated D1- and D2-agonist treatment via i.p. injection had no effect on markers associated with Ucp1-independent thermogenesis^32^ or dopamine clearance in iBAT (Fig. S4F).

### In vivo effects of dopamine receptor agonists on liver of wild type mice after daily i.p. injections for 7 days

**Figure 5:**
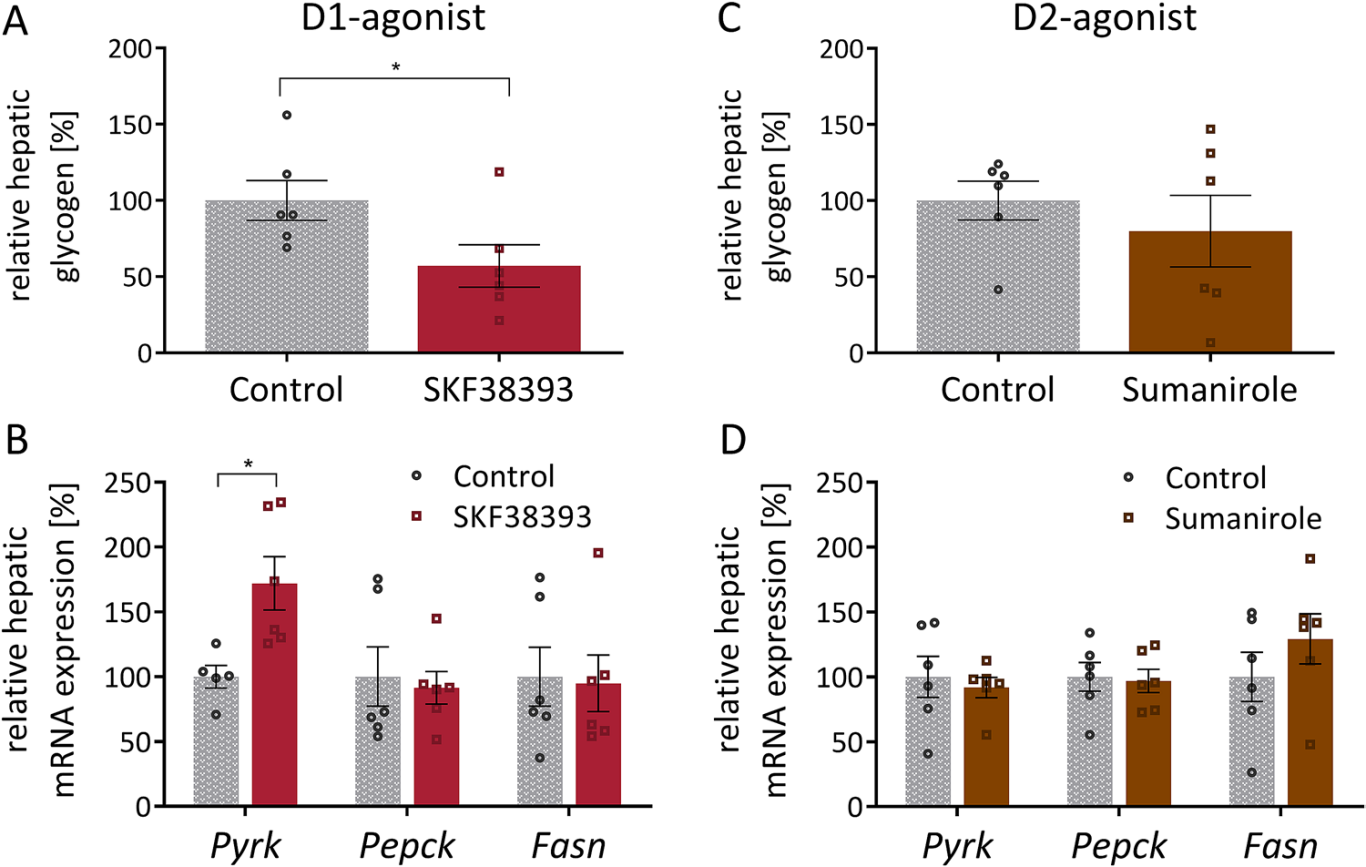
In vivo effects of dopamine receptor agonists on liver of wild type mice after daily i.p. injections for 7 days. Hepatic glycogen content (**A**,**C**) and relative hepatic mRNA expression of *Pyrk*, *Pepck*, and *Fasn* (**B**,**D**), determined after organ collection on day 7 of treatment with either D1-agonist SKF38393 (10 mg/kg) (left) or D2-agonist Sumanirole (3,2 mg/kg) (right). Data are expressed as mean ± SEM. Groups were compared using two-tailed t-tests. *P < 0,05; n = 6.

To dissect how the observed changes in fat depots affect other metabolic tissues, additional metabolic markers were analyzed in liver. In line with our observations in fat depots, indicating elevated metabolism, we found a significantly reduced amount of glycogen stored in the liver of mice treated with the D1-agonist (Fig. 5A), accompanied by a significant increase in mRNA expression of the rate-limiting enzyme in glycolysis, *Pyrk* (Fig. 5B), indicating increased glucose consumption in the liver. The D2-agonist had no significant effect on any of these parameters (Fig. 5C,D).

## Discussion

We investigated the role of dopamine receptor D1- and D2-agonists in the direct activation of iBAT thermogenesis in mice. Our results challenge the recent suggestions of tissular dopamine receptors being biologically relevant in BAT thermogenesis activation, as we have shown that acute peripheral administration of a D1-agonist increased iBAT temperature only for an extremely short and transient period briefly after injection, while repeated daily administration did not cause a sustained effect on iBAT temperature.

We observed rapid, yet very brief, acute effects of the D1- and D2-agonist on iBAT temperature in mice. According to their respective nature of being G_s_ or G_i_ protein-coupled receptor agonists^24,25^, the D1-agonist increased, while the D2 agonist decreased iBAT temperature. However, neither effect lasted until the end of the hour-long measurement and persisted only for 15 or 5 min, respectively. While this effect roughly follows the temporal dynamics previously described for cAMP levels in brown adipocytes, BAT, and the vasculature of BAT after NE administration^33–35^, it is not as sustained as the effect observed after NE stimulation. Likewise, when treated with dopamine, no effect on iBAT temperature was observed. This indicates that the pharmacological stimulation of a single receptor subtype (D1 or D2) may yield only a minor transient effect, which does not occur when stimulating both receptors simultaneously with the endogenous ligand dopamine.

After 1 week of repeated D1-agonist administration—in spite of elevated cAMP in iBAT—neither *Ucp1* nor iBAT temperature were increased. This is contrary to the well-known effect of an NE-induced cAMP increase^36^ and suggests differences in the downstream signaling cascade that, in case of the D1-agonist, do not further promote thermogenesis. The lack of downstream signaling could be caused by the biased agonism of SKF38393, namely G-protein signaling in absence of β-arrestin recruitment^37^, which can prevent late signaling and therefore avert sustained thermogenesis^38,39^. Moreover, the brief, acute changes in iBAT temperature could be indirect consequences of the D1- and D2-agonist affecting the vascular system^40^, either via peripheral action or via centrally mediated effects^41^.

The possibility of indirect action is supported by the lack of significant direct effects of the D1- or D2-agonist on mRNA expression of key thermogenic markers *Ucp1* and *Dio2 *ex vivo in isolated mouse iBAT explants. This was surprising, as it seems to contradict the recent suggestion of dopamine receptors D1 and D2 being present, and D1 being involved in brown adipocyte thermogenesis regulation in a murine in vitro model^28^. There are, however, several differences between the model system of iBAT explants, as used in our study, and immortalized brown adipocytes from mice^28^, e.g., in their degree of proliferation, multi-cellular interaction, and overall thermogenic capacity (given significantly lower expression of *Ucp1* mRNA in the immortalized cells as compared to explanted iBAT; unpublished observation). Our ex vivo model, which showed the expected BAT activation response upon stimulation with an ADRB3-agonist^14,42–44^, interestingly also showed a decrease of *Ardb3* mRNA upon stimulation with the D1-agonist. However, as this was not accompanied by activation of thermogenesis markers *Ucp1* or *Dio2*, it suggests only a negligible biological relevance for dopamine receptor D1 in the recruitment of BAT thermogenesis. While dopamine itself exerted similar effects on thermogenic markers *Ucp1* and *Adrb3* in iBAT explants, it additionally increased *Dio2* mRNA expression, suggesting a potential direct role of dopamine in tissular iBAT thyroid hormone production. Nevertheless, as we did not include data on UCP1 protein or DIO2 activity, we cannot fully exclude any additional non-transcriptional effects at present. Most importantly, however, dopamine—just like NE—does not exclusively bind to its designated receptors^19^, which allows the possibility that other receptors may contribute to this effect. Unfortunately, due to its extremely short half-life and rapid oxidation in vivo^45^, longterm studies as conducted with the individual agonists cannot be performed with dopamine in a similar setup.

Furthermore, to fully understand the direct role of dopamine receptors in BAT function, it has yet to be elucidated whether dopamine receptors are at all present on brown adipocytes. Our own dopamine receptor expression analyses in mice exhibited mRNA transcript levels of dopamine receptors D1–D5 in iBAT close to the detection limit (data not shown), which could also be caused by the remaining vasculature in the samples. Moreover, several commercially available antibodies for dopamine receptors D1 and D2 have recently been demonstrated to be unspecific^46^, thus questioning previous findings of D1 or D2 receptor protein in the tissue. Hence, it remains to be investigated whether dopamine receptors are truly present on brown adipocytes using more elaborate quantification techniques, e.g., on a single cell level.

Despite the lack of BAT thermogenesis activation, repeated D1-agonist administration still increased metabolic turnover in vivo, as evidenced by the reduced fat depot weights and lower hepatic glycogen levels. Ironically, this metabolic activation by the D1-agonist may even prevent activation of BAT thermogenesis in vivo, as it elevates obligatory thermogenesis, thus attenuating facultative BAT thermogenesis. Accordingly, emission of surplus heat would be probable, e.g., via tail vasodilation, which constitutes a major thermoregulatory mechanism in rodents^47,48^. This is supported by our finding of an increased tail base temperature, while core body temperature remained unaltered. The elevated tail base temperature could also result from D1-agonist-induced vasodilation^49–51^; however, if vasomotor-mediated tail heat loss were the primary effect, iBAT thermogenesis would be stimulated in order to compensate the heat loss and prevent a consequent decrease of core body temperature^48^—two effects not observed in our animal model.

Another possible mechanism could involve increased skeletal muscle activity, e.g., by enhanced locomotion as observed previously in D1-agonist-treated mice^52,53^ or by increased excitation of skeletal motoneurons^54^. This would subsequently lead to an elevated metabolic demand, which may be reflected by the reduced hepatic glycogen stores in our D1-agonist treatment group. Further studies, dissecting the role of dopamine receptors in muscle metabolism, are therefore warranted.

The lack of effects of the D2-agonist on iBAT thermogenesis shown in our study seemingly contradicts a recent study, where hypothalamic D2-agonist administration decreased body weight and stimulated iBAT activity in rodents^55^. However, in this study the effects of the D2-agonist were demonstrated to be mediated via centrally initiated activation of the sympathetic nervous system. Together with the low transport of the agonist across the blood brain barrier^56^, these results complement our findings suggesting that D2 receptors play a role in the central control of BAT thermogenesis, but not directly in BAT.

Overall, based on the lack of pronounced effects observed upon treatment with the D1- and D2-agonist in our study, we conclude that dopamine receptors D1 and D2 are unlikely to play a major physiological role in the peripheral regulation of BAT thermogenesis in mice. However, this does not fully exclude a potential role for dopamine itself in BAT thermogenesis activation^57^, as dopamine can also activate adrenoceptors—albeit less efficiently than NE^19,58^. Due to the extremely short half-life and rapid oxidation of dopamine after injection, this would, however, be difficult to test directly in a similar in vivo experiment and therefore needs to be studied separately. Taken together, our findings suggest that dopamine receptor D1- and D2-agonists might not constitute a promising therapeutic approach for peripherally inducing BAT thermogenesis for treatment of obesity or associated metabolic disorders.

## Methods

### Animals

All studies were carried out with 10–12 weeks old male wild type C57BL/6NCrl mice, purchased directly from Charles River, Germany. Upon arrival, the animals were housed in groups (except for acute treatment experiments with only one animal per cage), had one week to acclimate to the facility with 23 ± 1 °C, a constant 12 h light–dark cycle, and *ad libitum* access to food (standard diet 1314, Altromin) and water. All animal procedures were approved by the Ministerium für Energiewende, Landwirtschaft, Umwelt, Natur und Digitalisierung (MELUND) Schleswig–Holstein, Germany. All methods were performed in accordance with relevant guidelines and regulations.

### Substances

D1-agonist ( ±)-SKF-38393 hydrochloride (CAT# D047, Sigma), D2-agonist Sumanirole maleate (CAT# 2773, Tocris Bioscience), dopamine hydrochloride (CAT# ab120565, Abcam), ADRB3-agonist CL316,243 hydrate (CAT# C5976, Sigma), and NE compound “Arterenol” (CAT# 03870227, Sanofi) were solubilized to make identical stock concentrations in water or saline for ex vivo or in vivo studies, respectively. Final dilutions were freshly made in complete culture medium or saline for ex vivo or in vivo studies, respectively.

### Direct treatment of iBAT explants

The protocol for iBAT explant organ culture and medium composition was adapted from previous studies^59,60^. Briefly, iBAT was excised from 8 mice (12 weeks of age) per experiment, cut into four pieces and transferred into basal differentiation medium (Medium 199, 2% BSA, 25 mM HEPES, and 0,25 mg/ml gentamycin sulfate). Pooled iBAT was minced into pieces of 1–2 mm in diameter, rinsed with DPBS, and distributed evenly into 12-well culture plates (~ 10 mg tissue/well) filled with 1 ml complete differentiation medium (basal differentiation medium with 0,7 nM insulin, 2 nM 3,3′,5-triiodothyronine (T3), 2,5 nM dexamethasone, and 150 µM L-ascorbic acid). Explants were synchronized in 37 °C and 5% CO_2_ for 24 h and then rinsed with DPBS, which was subsequently replaced by culture medium with added treatment substances (3 technical replicates per condition). Finally, explants were incubated in 37 °C and 5% CO_2_ for 24 h, rinsed with DPBS, collected, snap frozen, and stored at − 80 °C until further processing.

### Single injection (acute) in vivo experiment

To study acute effects of the D1- and D2-agonist on iBAT thermogenesis, mice (n = 6) received saline, the D1-agonist SKF38393 (10 mg/kg), D2-agonist Sumanirole (3.2 mg/kg), or dopamine (100 µg/kg) via intraperitoneal (i.p.) injection. One animal was injected with 1 mg/kg NE (s.c.) as a positive control reference. Infrared videos (1 picture/s) were shot using a VarioCAM hr head (InfraTec). First, 10 min were filmed for baseline measurements. Subsequently, animals were briefly restrained for i.p. injection, released back into the cage, and filmed for another 60 min. iBAT temperature of one representative picture/min was scored using the IRBIS3 software (InfraTec). Changes in iBAT temperature were calculated by subtracting the 10 min baseline average score from 5 min interval average scores.

### Repeated injection (chronic) in vivo experiment

To study chronic effects of the D1- and D2-agonist on iBAT thermogenesis, mice were monitored for two weeks. Measurements of the first week were collected for baseline data, while measurements of the second week were collected under treatment. During the second week, animals received saline (n = 6) or one of the agonists (n = 6) via daily i.p. injection (10 mg/kg D1-agonist SKF38393 or 3.2 mg/kg D2-agonist Sumanirole, both half-life > 4 h^31,61,62^); each treatment had its own independent saline control group.

Infrared photo thermography was done with a FLIR T335 (FLIR Systems) infrared photo camera, on the last two days of both, the first (baseline) and second (treatment) week. iBAT and tail base temperature was scored using the FLIR Tools Software (FLIR Systems). The scored temperature was normalized to rectally measured core body temperature (RET-3 Rectal Probe for Mice, BAT-12 Microprobe Thermometer; both Physitemp Instruments).

Simultaneous measurements of heart rate (pulse), systolic blood pressure (SBP), diastolic blood pressure (DBP), and mean arterial pressure (MAP) were recorded using the non-invasive SC1000 Single Chanel System (Hatteras Instruments). An automated sequence of 15 measurements was conducted in each session. The mean of at least 12 values for each parameter from sessions on two consecutive days was used for statistical analyses.

### Quantitative real-time PCR (qRT PCR)

Isolation of total RNA and transcription into cDNA were done according to manufacturer’s instructions for the RNeasy Mini/RNeasy Lipid Tissue Mini Kit (CAT# 74104/74,804, both Qiagen) and RevertAid First Strand cDNA Synthesis Kit (CAT# K1622, Thermo Scientific), respectively. Genes of interest were analyzed by qRT PCR. Fast Start Universal SYBR-Green Master (ROX) (CAT# 4913914,001, Roche) was used for all qRT PCR experiments, according to the manufacturer’s instructions. Primers were used in final concentrations of 50 nM. Primer sequences (5′-3′) are listed below.

**Table:** 

*Ucp1*	fw: ACT CAG GAT TGG CCT CTA CG	rev: CCA CAC CTC CAG TCA TTA AGC
*Dio2*	fw: ATG GGA CTC CTC AGC GTA GAC	rev: ACT CTC CGC GAG TGG ACT T
*Adrb3*	fw: AGA AAC GGC TCT CTG GCT TTG	rev: TGG TTA TGG TCT GTA GTC TCG G
*Pyrk*	fw: TCA AGG CAG GGA TGA ACA TTG	rev: CAC GGG TCT GTA GCT GAG TG
*Pepck*	fw: ATC TTT GGT GGC CGT AGA CCT	rev: GCC AGT GGG CCA GGT ATT T
*Fasn*	fw: GGA GGT GGT GAT AGC CGG TAT	rev: TGG GTA ATC CAT AGA GCC CAG
*Gamt*	fw: CAC GCA CCT GCA AAT CCT G	rev: TAC CGA AGC CCA CTT CCA AGA
*Gatm*	fw: GCT TCC TCC CGA AAT TCC TGT	rev: CCT CTA AAG GGT CCC ATT CGT
*Phospho1*	fw: ATG AGC GGG TGT TTT CCA G	rev: ATC GAA GTC GAA GGT GAG GAG
*Serca2*	fw: TCC GCT ACC TCA TCT CAT CC	rev: CAG GTC TGG AGG ATT GAA CC
*Slc6a8*	fw: GTC TGG TGA CGA GAA GAA GGG	rev: CCA CGC ACG ACA TGA TGA AGT

### Western blot

Separation of isolated proteins was achieved by SDS-PAGE with 12% gels (TGX Stain-Free FastCast Acrylamide Solutions, CAT# 1610185, Bio-Rad) and 20 µg total protein per lane. Total protein was detected for semi-quantification using Stain-Free imaging according to the manufacturer’s instructions (Bio-Rad). Wet transfer was conducted to a PVDF membrane. Membranes were incubated with primary antibodies UCP1^63^, OXPHOS Rodent WB Antibody Cocktail (CAT# 45-8099, Thermo Fisher), DAT (dopamine transporter) (CAT# ab184451, Abcam), MAO-A (monoamine oxidase A) (CAT# ab126751, Abcam), or SERCA2 (sarco/endoplasmic reticulum Ca^2+^-ATPase) (CAT# 4388, Cell Signaling) overnight at 4 °C. Incubation with a secondary antibody (Polyclonal Goat Anti-Rabbit Ig/HRP, CAT# P0448, Dako; Polyclonal Goat Anti-Mouse Ig/HRP, CAT# P0447, Dako) was performed for 1 h at room temperature, before bands were detected with Clarity Max Western ECL substrate (CAT# 1705062, Bio-Rad) and a ChemiDoc Touch Imaging System (Bio-Rad). Semi-quantitative analysis was performed in Image-Lab (Bio-Rad). Target protein abundance was normalized to total protein.

### Hepatic glycogen content

Hepatic glycogen was determined as published by Vujovic et al.^64^, with minor modifications. Specifically, after 2 × volume ethanol (> 95%) was added to 50 µl supernatant, samples were incubated at 4 °C overnight. Furthermore, after re-suspension in 80 µl Lugol Reaction Mix, sample reaction developed for 10 min before OD_600_ was measured (75 µl/well in a 96-well plate). Values were normalized to the amount of tissue used.

### ELISA and enzyme activity

cAMP concentration and lipase activity in fat depots of mice were measured by following the manufacturer’s instructions for the Non-Acetylation EIA Procedure of the Amersham cAMP Biotrak Enzymeimmunoassay (EIA) System (CAT# RPN225, GE Healthcare) and Lipase Activity Kit colorimetric (CAT# ab102524, Abcam), respectively.

### Histological staining

Hematoxylin and eosin (H&E) staining was performed on 5 µm histological sections of paraffin embedded tissue. Sections were dewaxed in xylene (2 × 15 min), hydrated in descending ethanol concentrations (100% 2 × 5 min, 96% 5 min, 80% 5 min, 70% 10 min), and washed in de-ionized water (6 min). Nuclear staining was attained in a hematoxylin bath (4 min), followed by flowing tab water (15 min) and a bath in de-ionized water (2 min). Next, non-nuclear staining was achieved in an eosin bath (2 min) and de-ionized water (10 s). Lastly, sections were dehydrated in ascending ethanol concentrations (70% 2 × 10 s, 80% 15 s, 96% 20 s, 100% 2 × 30 s) and cleared in xylene (2 × 15 min). Specimens were mounted with Pertex (Medite Medical) and covered with a glass cover slip.

### Statistics

Values are presented as mean ± SEM. Statistical analyses were performed using Prism6 (Graphpad Software) and considered significant with *P < 0,05; **P < 0,01; ***P < 0,001; and ****P < 0,0001. Statistical tests were applied as indicated in figure legends. Grubb’s outlier test was performed with α = 0.01 for ex vivo and α = 0.05 for in vivo experiments. Statistically significant outliers with significant impact on results were excluded from graphs and calculations.

## Supplementary information

Supplementary Information

## Data Availability

The data that support the findings of this study are available from the corresponding author upon reasonable request.
