# Supplementary material for: Dopamine receptor D1- and D2-agonists do not spark brown adipose tissue thermogenesis in mice
Source: Sci Rep. 2020 Nov 19;10:20203. doi: 10.1038/s41598-020-77143-6 (PMC7677542; doi:10.1038/s41598-020-77143-6)
Supplement: Supplementary file 1 — Supplementary Information [file 41598_2020_77143_MOESM1_ESM.pdf]

Dopamine receptor D1- and D2-agonists do not spark brown adipose tissue thermogenesis in mice

Francesca-Maria Raffaelli[a], Julia Resch[a], Rebecca Oelkrug[a], K. Alexander Iwen[b#], Jens Mittag[a#*]

Supplementary Figure S1

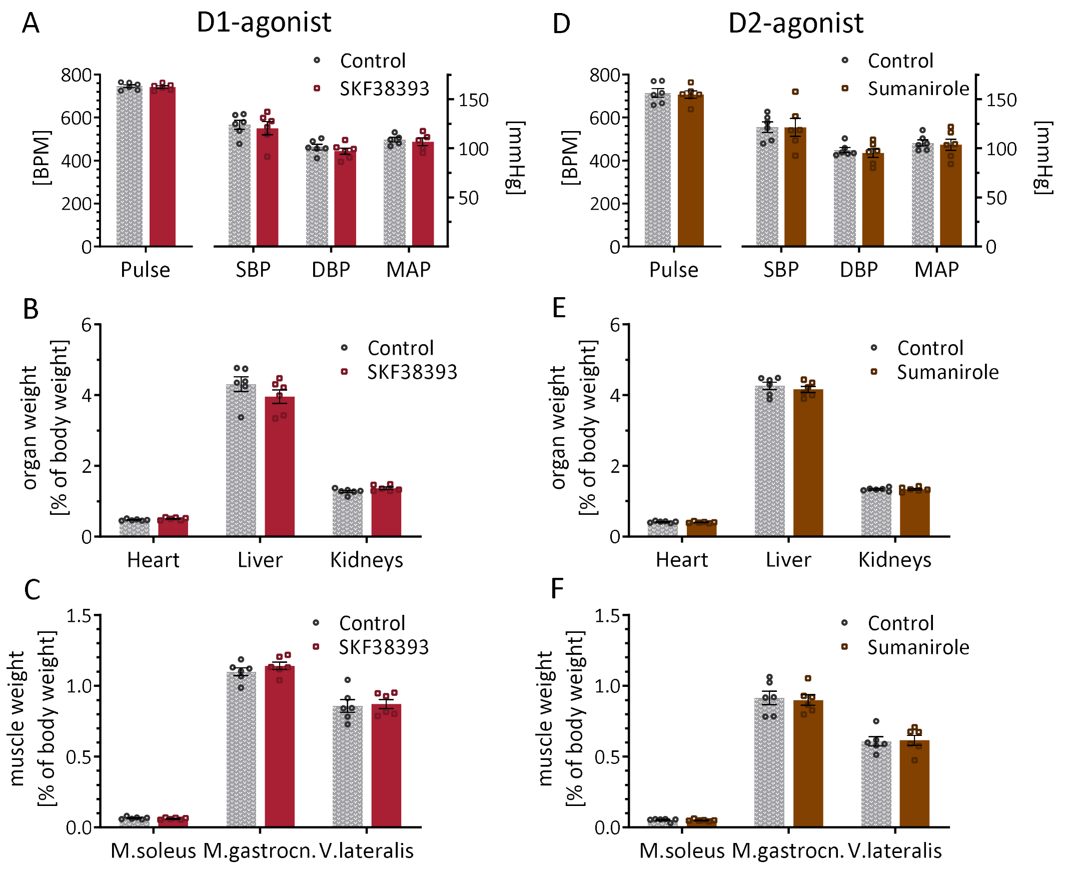

Fig. S1: ***In vivo*** **effects of dopamine receptor agonists on pulse, blood pressure, organ, and muscle weights of wild type mice during daily i.p. injections after 7 days.**
Pulse, systolic blood pressure (SBP), diastolic blood pressure (DBP) and according mean arterial pressure (MAP) of mice treated D1-agonist SKF38393 (10 mg/kg) (**S1A**) or D2-agonist Sumanirole (3,2 mg/kg) (**S1D**). Organ (**S1B, E**) and muscle weights (**S1C, F**), determined after organ collection on day 7 of treatment with either D1- (**left**) or D2-agonist (**right**). Data are expressed as mean ± SEM. Groups were compared using two-tailed t-tests.

Supplementary Figure S2

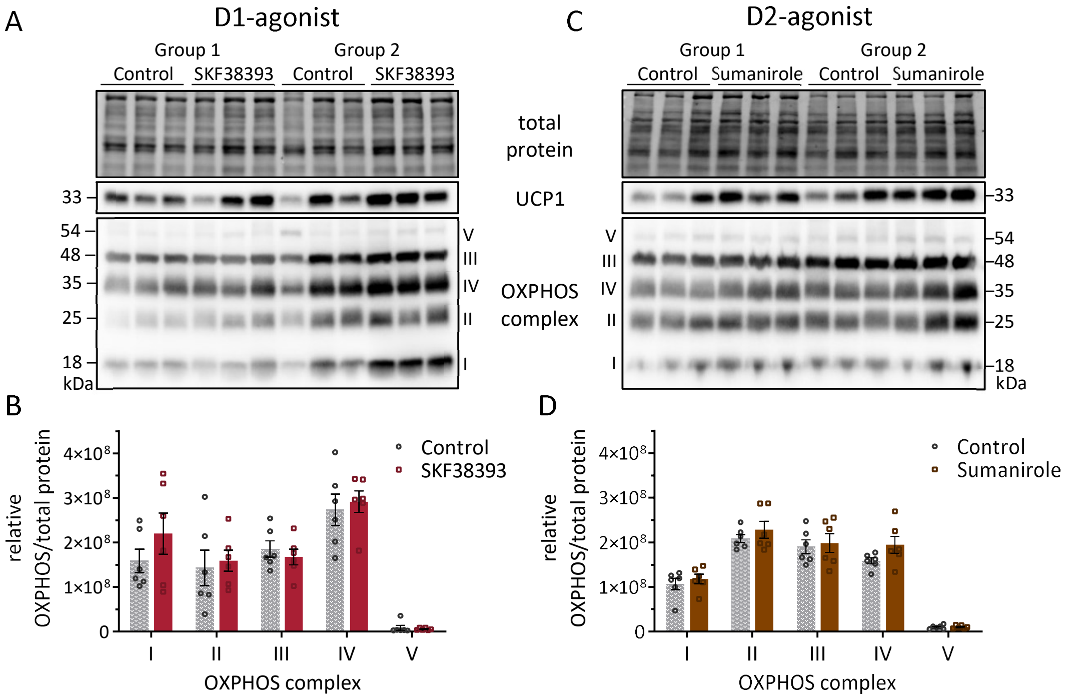

Fig. S2: ***In vivo*** **effects of dopamine receptor agonists on protein expression in iBAT of wild type mice after daily i.p. injections after 7 days.**
Bands show total protein load, UCP1, and OXPHOS complexes in iBAT, acquired by Western blot analysis **(S1A, C)**. Relative protein abundance of OXPHOS protein complexes I-V **(S1B, D)**. Data are expressed as mean ± SEM. Groups were compared using two-tailed t-tests. n=6

Supplementary Figure S3

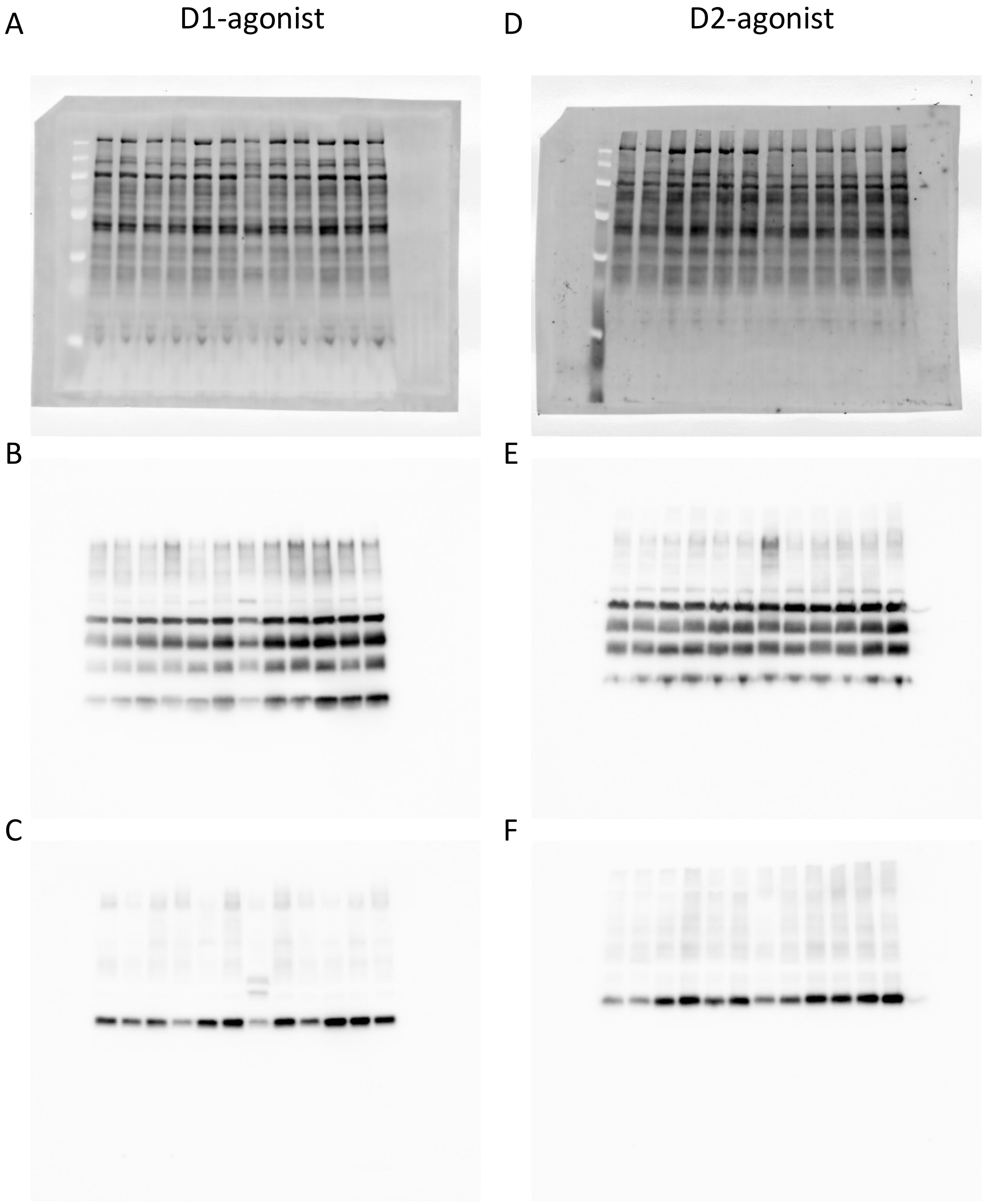

Fig. S3: Original-sized Western blot images.
Original, uncropped Western blot images for total protein **(S3A, D)**, OXPHOS complexes **(S3B, E)** and UCP1 **(S3C, F)** in iBAT of mice treated with the D1-agonist **(S3A-C)** or the D2-agonist **(S3D-F)**.

Supplementary Figure S4

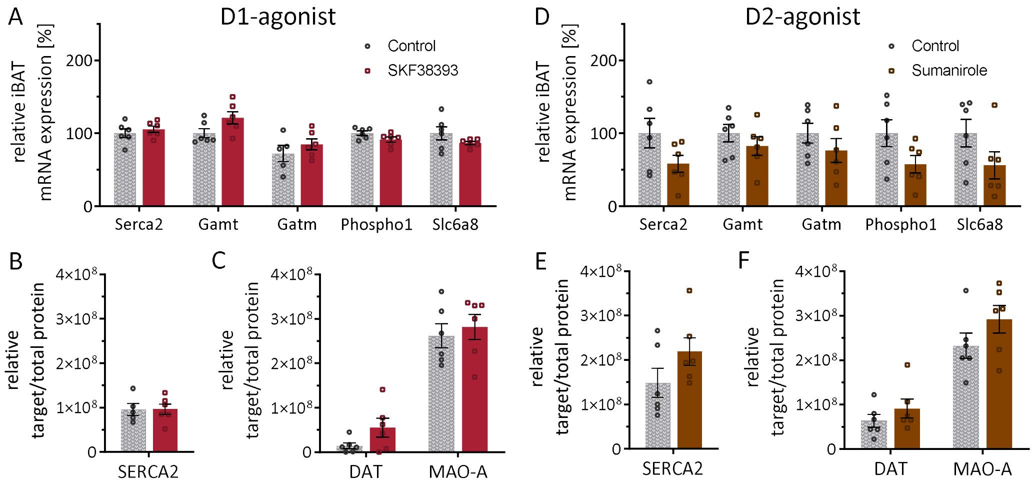

Fig. S4: Markers of alternative (*Ucp1*-independent) thermogenesis activation and dopamine clearance
Relative iBAT mRNA expression of markers for alternative thermogenesis activation **(S4A, D)**. Relative iBAT protein abundance of alternative thermogenesis activation marker SERCA2 **(S4B, E)**, and dopamine clearance-associated proteins DAT and MAO-A **(S4C, F)**. Data are expressed as mean ± SEM. Groups were compared using two-tailed t-tests. n=6
